# The relationship between expelled eggs, morbidity and age in a *Schistosoma mansoni* endemic setting in Uganda: Implications for current elimination policies

**DOI:** 10.1371/journal.pntd.0012750

**Published:** 2025-09-03

**Authors:** Rivka M. Lim, Ruhi Lahoti, Jessica Clark, Moses Arinaitwe, Victor Anguajibi, Sergi Alonso, Andrina Nankasi, Fred Besigye, Alon Atuhaire, Amy B. Pedersen, Joanne P. Webster, Poppy H. L. Lamberton

**Affiliations:** 1 Institute of Evolution and Ecology, School of Biological Sciences, Ashworth Laboratories, University of Edinburgh, Edinburgh, United Kingdom; 2 School of Biodiversity, One Health and Veterinary Medicine, University of Glasgow, Glasgow, United Kingdom; 3 China-Uganda Friendship Hospital, Kampala, Uganda; 4 Global Business School for Health, University College London, London, United Kingdom; 5 Vector Borne and Neglected Tropical Diseases Control Division, Ministry of Health, Kampala, Uganda; 6 Department of Pathobiology and Population Sciences, Royal Veterinary College, University of London, Herts, United Kingdom; Weill Cornell Medical College, UNITED STATES OF AMERICA

## Abstract

Direct morbidity assessments are rarely included in monitoring and evaluation of *Schistosoma mansoni* mass drug administration programmes. This is despite morbidity reduction being the leading objective of control and elimination as a public health problem in the World Health Organization (WHO) targets. Instead, the number of eggs-per-gram (EPG) of faeces are used as a morbidity proxy. Furthermore, current WHO guidelines use infection intensity thresholds to determine where and when MDA is to be implemented. However, recent work has begun to question this assumption of a direct association between infection intensity in intestinal schistosomiasis and host morbidity. Here we aimed to examine the potential association between *S. mansoni* infection intensity and morbidity from pre-school-aged children (PSAC) through to elderly individuals, living in Bugoto, Uganda. Prevalence and intensities of *S. mansoni* infection were diagnosed by Kato-Katz and point-of-care circulating cathodic antigen tests (POC-CCAs) in 287 individuals aged 3–74 years, from Bugoto, Uganda. In addition to data on anaemia and self-reported symptoms, abdominal ultrasound examinations were conducted to identify liver parenchyma image pattern (IP), portal vein dilation (PVD) and left parasternal line (PSL) enlargement. Malaria status was determined using rapid diagnostic testing. Generalised additive models estimated associations between morbidity outcomes and infection intensity/presence, diagnostic method, co-infections, age and sex. The prevalence of positive IP scores, dilated PVD, enlarged PSL and anaemia were 9%, 34%, 33% and 13% respectively. Neither *S. mansoni* infection intensity or status were significantly associated with PVD, PSL, or anaemia. Age was the most consistent predictor of morbidity, with the highest burden of PVD, PSL and anaemia in PSAC. Malaria infection was also positively associated with PVD and anaemia. A positive POC-CCA predicted only self-reported blood in stool. Our findings add to growing evidence that current infection intensity is an inappropriate proxy for schistosomiasis morbidity, urging a revaluation of tools and targets. The observed prevalence of morbidities in PSAC evidence a need to elucidate the impact of less-specific morbidities, past *S. mansoni* and other parasitic infections on host health, and adds urgency to the on-going roll out of treatment to this age group.

## Author summary

Schistosomiasis is a major parasitic disease that affects millions of people worldwide, especially in sub-Saharan Africa. Monitoring and control programmes for intestinal schistosomiasis typically rely on counting parasite eggs in stool as a proxy to measure disease presence and severity, but recent evidence questions whether this method accurately reflects the real health impact of the disease. Here we examined 287 individuals in a high-risk community in Uganda, aged 3–74 years, using both stool and urine tests to detect *Schistosoma mansoni* infection. We also used abdominal ultrasound to assess liver damage and other health complications caused by the parasite and used rapid testing of participants’ blood to diagnose malaria and anaemia. Surprisingly, we found that even at very low *S. mansoni* infection levels, young children had significant liver-related complications and anaemia. Additionally, current malaria infection was strongly linked to anaemia and liver abnormalities, indicating the complexity of co-morbidity in co-endemic areas. Our findings suggest that the current World Health Organization thresholds for defining disease severity does not provide an accurate picture of the true burden of schistosomiasis, particularly in young children and in regions of co-endemicity with malaria. This research highlights the urgent need for new methods to assess the impact of the disease and the inclusion in treatment programs for young children.

## Introduction

Schistosomiasis is a parasitic disease caused by trematodes of the *Schistosoma* genus. It is a substantial cause of global morbidity, with the highest disease burden found within sub-Saharan Africa [1]. *Schistosoma mansoni* is one of the key species responsible for the hepato-splenic and intestinal forms of the disease in humans, and infection is acquired by contact with freshwater infested with cercariae larvae that are shed from intermediate *Biomphalaria spp* snail hosts*.* Cercariae penetrate human skin, enter the bloodstream and migrate through the heart and lungs to the liver where they mate and mature [2,3]. Afterwards, most adult *S. mansoni* worms travel to the portal vein and mesenteric venules, where adult worm pairs are estimated to produce up to 300 eggs per day [4]. These laterally spined eggs are either shed from the body in the faeces, which drives onward transmission, or become trapped in host tissues. This can cause inflammation, granulomas, damage, and scarring, which can obstruct the veins surrounding the liver and cause hepatomegaly, portal hypertension, liver dysfunction and periportal fibrosis [5,6]. Infection can also cause non-specific symptoms such as abdominal pain, diarrhoea, blood in the stool, malnutrition, anaemia, stunting, and cognitive dysfunction [6,7].

The Niamey Protocol was developed to standardise the ultrasound-based evaluation of morbidity, focusing on detecting organ dysfunction, periportal fibrosis, portal hypertension, hepatosplenomegaly and ascites [8]. Other less specific diagnostic methods for morbidity include faecal occult blood point-of-care tests, visual identification of blood in stool, diarrhoea, self-reported abdominal pain and blood tests for anaemia [9]. Periportal fibrosis as diagnosed by the Niamey Protocol is aimed to be specific to *S. mansoni-*associated fibrosis. In contrast, less specific tests including liver and spleen enlargement may complicate the accurate attribution of morbidity causes. This limitation is particularly relevant in regions co-endemic for other infectious diseases such as malaria and soil-transmitted helminths (STHs) which can present with similar morbidity outcomes [10]. Additionally, these morbidity data are not typically collected during routine surveillance, monitoring and evaluation activities. Instead, faecal egg counts indicating current infection intensity are used as a proxy for morbidity with those shedding a high number of eggs assumed to be most at risk for morbidity [11–13].

Faecal egg counts (quantified as eggs-per-gram (EPG) of stool) and therefore diagnosis of *S. mansoni* is most commonly conducted using light microscopy and the Kato-Katz thick smear method [14]. For operational purposes, The World Health Organization (WHO) classifies infections as light, moderate and heavy (1–99, 100–399 and ≥400 EPG, respectively) [15]. However, the Kato-Katz method lacks sensitivity, particularly in low prevalence regions and when numbers of expelled eggs are low [16]. There can also be significant variation in day-to-day egg shedding rates from the same individual [17]. In 2017 the WHO endorsed the use of point-of-care circulating cathodic antigen (POC-CCA) tests for *S. mansoni* diagnosis [18]. These tests detect antigens regurgitated by feeding adult worms which are excreted in an infected person’s urine [18] and have higher sensitivity than Kato-Katz when detecting low-intensity infections [16], but concerns about the specificity of POC-CCA have emerged. A study by Clark et al. (2022) used Bayesian latent class analysis to assess the relationship between EPG, POC-CCA, and true infection status, and determined that using a POC-CCA score of G3 [19] offered a false positive rate of ~10–18% and high sensitivity (~90%) [20]. However, whilst statistical correlations between egg counts and POC-CCA exist [16,21,22] the two diagnostics measure different aspects of infection: egg output versus worm presence, and therefore may not align in their relationship to morbidity.

The main method of schistosomiasis control recommended by the WHO is mass drug administration (MDA), where at-risk communities are treated with the anthelminthic drug praziquantel, regardless of individual current infection status [23,24]. Due to the observed relationship between infection intensity and morbidity prevalence at the start of MDA, the initial objective of these programmes was reduce infection intensities as measured by Kato-Katz as a proxy indication for the reduction in morbidity prevalence [23,25]. School-aged-children (SAC) were therefore targeted for treatment as they typically harbour the highest infection intensities, to lower risk of morbidities later in life and because this age group is easier to access through schools [23]. However, whilst praziquantel treatment is effective at killing adult worms [22], it is not effective on juvenile worms and does not prevent reinfection [22]. As a result, heavy infections can rapidly develop/return post-treatment with the potential to cause further morbidity. Additionally, disease symptoms and associated morbidity may not be reversible with treatment especially if the infection is chronic or severe [26–28]. Furthermore, recent evidence challenges the use of current *S. mansoni* infection intensity as predictive of morbidity. A multi-country cohort study of SAC using ultrasound, clinical findings, and self-reported symptoms found no consistent association between WHO-defined infection intensity categories and morbidity markers [29]. An earlier comparative study from Egypt and Kenya also found no clear epidemiological association between infection intensities and morbidity markers such as periportal fibrosis or portal hypertension, despite substantial differences in infection intensity and prevalence of these conditions between the two regions [30]. Moreover, a recent systematic review and meta-analysis found an association between liver morbidity and current *Schistosoma* infection caused by intestinal species prior to the implementation of nationwide MDA programmes in 2003, however this association diminished in the post-MDA era [31].

Despite this lack of contemporary evidence to support the relationship between infection intensity and morbidity, the 2021–2030 Neglected Tropical Disease Roadmap which has targeted schistosomiasis for Elimination as a Public Health Problem (EPHP), has set the threshold for achieving this as <1% of heavy infections (>400EPG) in SAC in a community. This approach therefore continues to use infection intensity as a proxy metric for morbidity control [32]. Key to achieving EPHP is the inclusion of all children over two years of age (supported by the development and deployment of a paediatric formulation of praziquantel [33]), and an increase to biannual treatment in areas of high endemicity or persistent hotspots [34]. However, as control efforts advance there is growing concern that current morbidity definitions may not adequately take into consideration the differences between heavy and moderate infection intensities [35,36], or account for the impact of decades of MDAs, that may have mitigated disease progression leaving individuals with underlying morbidity or undetectable infections which could be missed by current guidelines. One solution posed by many is the redefining of targets based on overall infection prevalence, rather than heavy-intensity infections alone [35–37], though it is also unclear whether this would be any more suitable.

Disentangling the drivers of morbidity across different age groups remains a challenge for how best to optimise resource allocation and treatment strategies. Whilst previous studies have largely focused on SAC, there is a notable gap in research that examines morbidity and the influence of co-endemic infections on morbidity across the full spectrum of age groups in the same community, especially for PSAC. Additionally, although both the Kato-Katz method and the more sensitive POC-CCA diagnostic have been employed in past studies, there is a lack of research that systematically applies both diagnostics, detecting worm antigens and eggs simultaneously alongside multiple morbidity predictors, both specific and nonspecific, across all age groups.

Therefore, the objective of this study was primarily to understand whether infection intensity is associated with morbidity at a given time point, and whether egg- and antigen-based diagnostics have differing relationships with morbidity. We further examined the age-related distributions of morbidities, potentially reflecting the impacts of long-term chronic exposure. Finally, we sought to examine the relationship between *Schistosoma*-associated morbidity and the presence of current infections, including *S. mansoni*, *Plasmodium,* or STH.

## Methods

### Ethical Statement

Ethical clearance was granted by the Uganda National Council for Science and Technological Social Sciences (reference: 2193), the Ugandan Ministry of Health Vector Control Division Research Ethics Committee (reference: VCDREC/062) and the University of Glasgow College of Medical, Veterinary and Life Sciences (reference:200160068). Informed written consent was gathered prior to any data collection from all participants over the age of 18 by either signature or thumb print, whilst parents or legal guardians gave written consent via signature or thumb print for those under 18 years old, and informed verbal assent was also obtained from all children between eight and 18 years old. All participants were made aware that they were allowed to opt out of the study at any point without losing any benefits they were entitled to during the study, and it would not affect their standard of care. If any participant was found to have severe morbidity they were referred to the local hospital.

### Study area

This study was carried out in May 2022 in Bugoto community, which comprises of two villages: Bugoto A and Bugoto B, located on the north shores of Lake Victoria, Mayuge District, Eastern Uganda. These communities and others along the shores of Lake Victoria are highly endemic for *S. mansoni* [38–40] and these small rural village communities are mostly involved in small scale agriculture and fishing, where daily interactions with the lake are the norm [38]. Annual MDA with praziquantel in SAC has been taking place in these villages since 2003. Community-wide (SAC and adults only, not including PSAC) launched in 2004 and in 2019 this increased to community-wide biannual (SAC and adults only) [25,38,41]. This area is also highly endemic for malaria infection caused mainly by *Plasmodium falciparum*, with a prevalence of 21% in children <5 years old in the sub-region of Busoga, which includes the study community of Bugoto [42,43]. Malaria control measures include community-wide distribution of insecticide-treated nets every three years, health-centre-based treatment for those seeking it and integrated community case management (ICCM) of malaria in children, which is test and treat for suspected malaria patients [42]. Annual MDAs also include treatment of 400mg albendazole for STH, which are often co-endemic [39].

### Data collection

Data were gathered over a two-week period in the wet season and just prior to the annual national MDA when people are expected to have the highest infection intensities. All members of the village apart from children under 2 years old (due to this being before the very recent roll out of the paediatric formulation of praziquantel) were eligible to participate, although the youngest child that enrolled was 3 years of age, and Village Health Team members assisted with recruitment and informed consent. A formal power analysis was not conducted prior to the study due to the lack of existing values to effectively parameterise a power analysis across all morbidly markers and age groups. However, a comparable study assessing liver morbidity scores conducted in a nearby village with similar predicted *S. mansoni* prevalence estimated that a sample size of 156 was sufficient to explore infection–morbidity patterns [40]. Based on this and the aim of acheiving sufficient power for a larger range of age groups, we aimed for a larger sample size (~400), knowing that some participants would likely not complete all testing and to ensure the final sample would remain broadly representative of the community. This is approximately 11% of the total community which has an estimated 3500 residents [44]. According to the Uganda Population and Housing Census (2014), the age distribution in Mayuge District comprises approximately 20% preschool-aged children (<6 years), 34% school-aged children (6–14 years), and 46% adults (≥15 years), with females making up 52% of the population. Participants were randomly selected across these stratified age categories to reflect this age and sex structure. Our final sample included 287 individuals with a slightly higher proportion of females (63%) and a median age of 14 years and overall remained broadly reflective of the district population.

Parasitological, clinical and ultrasound measurements were performed in the field. Height, weight, age and sex of each participant was recorded alongside questions about schistosomiasis-related symptoms they had experienced in the last month, previously described in full [45]. Smartphones using ODK software were used to collect data.

Three stool samples from each participant were obtained on three separate consecutive days and duplicate Kato-Katz [14] were prepared per stool resulting in up to six egg counts per person. These smears were examined within one hour of preparation to quantify *S. mansoni, Ascaris lumbricoides*, *Trichuris trichiura*, *Enterobius vermicularis, Hymenolepis nana* and hookworm eggs. Individual infection, infection intensity (EPGs) and community infection prevalence were calculated. Urine samples were collected for POC-CCA cassette testing over three days (Schisto POC-CCA, ICT International, Cape Town, RSA) following the manufacturer’s instructions, and semiquantitative results (G1 to G10) were assigned using the G-score system [19]. Two POC-CCA batches were used (210811080 and 211110105) and for each batch reference standards of four concentrations of trichloroacetic acid-soluble fraction of *S. mansoni* adult worm antigen, containing ~3% CCA was run [46]. Malaria infection was determined using finger prick blood (Ag P.f/Pan Malaria Rapid Diagnostic test, Standard Diagnostics Inc.) [47], which can diagnose *Plasmodium* species in whole blood.

### Ultrasound examination

Each participant was examined in the supine position using the portable ultrasound machine, Aloka Prosound 2 (Hellige, Freiburg, Germany), equipped with curve-linear probe and variable frequency of 2.5-6MHz. These were performed by a single senior radiographer trained in the Niamey protocol [8] from the Ministry of Health in Uganda who was blinded to the infection status of the individual. The left liver lobe size was measured from the upper to the caudal margin in the left parasternal line (PSL) of the lobe to indicate for left liver lobe enlargement. Portal Vein Diameter (PVD) measurements and the presence of any ascites or collaterals was also recorded and were taken to gauge for risk of portal hypertension. Both the PSL and PVD measurements were standardised by height and compared to healthy Sudanese controls from a non-endemic population [8,48]. Liver parenchyma image pattern (IP) was designated a score from A-F, where A is classed as no morbidity, B is undetermined and C-F (and combinations thereof are scored, referencing the Niamey protocol). As we did not have data on the thickness of the walls and branches of the portal vein due to equipment constraints, we did not calculate a periportal thickening (PT score) as per Niamey protocol. The spleen was not measured as per instructions in the protocol for regions which are endemic for malaria, however if splenomegaly was observed the sonographer made a note of it in the participant’s record. Any participant observed to have severe pathology were referred to the appropriate health authority.

At the end of the study and in accordance with national guidelines all participants were given bread and juice and offered praziquantel at 40mg/kg body weight and 400mg albendazole. Those diagnosed as positive for malaria were offered dihydroartemisinin/piperaquine, the current WHO recommended treatment, given directly to them or the parents/legal guardians.

### Data processing

Data were exported to R version 4.2.2 where all data management and statistical analysis were executed. Seven age groups were established from the study population for descriptive analyses: i) 3 – 5 years, ii) 6 – 10 years, iii) 11 – 14 years iv) 15 – 20 years, v) 21 –30 years, vi) 31 – 40 years, and vii) > 40years and three age classes were also established: i) 3–5 years = PSAC, ii) 6–15 years = SAC, and iii) >15 years = adults.

To score morbidity in the participants we used a binary system. PVD was determined by comparing the PVD to height-matched healthy controls [8,48], a PVD score was given depending on if the PVD was normal size (score = 0) or dilated in which it was > 2 standard deviations (SD) above the healthy comparisons (score = 1). The left liver lobe measurement was also compared to healthy standards [8,48] if not enlarged (score = 0) or enlarged in which it was > 2 SD larger than healthy comparators (score = 1). Anaemia was categorised as either positive (score = 1) or negative (score = 0) for statistical modelling or normal, mild, moderate and severe when used as a descriptor, calculated as referenced by the WHO for Hb (g/l) measures at 1200 metres above sea level [49]. A score of 1 was given each time a participant answered yes to survey questions regarding symptoms which could be attributed to *S. mansoni* infection: diarrhoea, blood in stool, abdominal pain, pain when urinating, nausea, vomiting, headache, fever, dizziness, body swelling, chills, difficulty breathing, muscle pain, rash and/or weakness.

### Statistical analysis

#### Characterisation of *Schistosoma mansoni* prevalence, intensity, and liver morbidity in a highly endemic population.

Descriptive statistics were used to summarise participant demographics and prevalence data. Participant age, sex, the number of stool and urine samples and morbidity metrics were summarised using means, medians and proportions. Positivity was attributed when a participant was found to have at least one *Schistosoma* egg on any given day or was positive by POC-CCA with a score of G3 or more [20]. The overall prevalence for each group was then calculated by dividing the number of positive individuals by the total number of participants in the group. Prevalence was reported for both diagnostic methods. Intensity of infection was calculated for all participants as the arithmetic mean or the geometric mean of EPG, geometric mean was calculated by taking the natural logarithm of all non-zero values, computing the arithmetic mean of the logged values and exponentiating the result.

#### Modelling the association between *Schistosoma mansoni* mean infection intensity and morbidity.

The descriptive analyses indicted that morbidity varied non-linearly with age. We therefore applied a Generalized Additive Model (GAM) to allow for the non-linear effect of age. Each morbidity marker (PVD, PSL, anaemia and 15 different self-reported symptoms) was modelled as a binary response variable using a binomial distribution. The predictors included age as a continuous variable with an appropriate smoothing term (described below), mean infection intensity of *S. mansoni* (as an additional smooth term), hookworm infection status, malaria infection status and sex (all as categorical parametric terms). To select the optimal smoothing function, we compared cubic regression splines, thin plate regression splines, and P-splines using maximum likelihood estimation and compared using the Akaike Information Criterion (AIC), with the model that yielded the lowest AIC selected. This model was then used for different smoothing parameters (sp) at values of 0.1, 1, and 2 or restricted maximum likelihood (REML) and again the model with the lowest AIC was selected. Model diagnostics were performed using the Dharma package (version 0.4.6), checked with the gam.check function from mgcv package (version 1.9-1) with adjustments made if significant issues were detected.

#### Modelling the association between *Schistosoma mansoni* infection status and morbidity.

Here we also used GAMs to model *S. mansoni* infection status and morbidity, with age as the only smoothing variable, and hookworm, malaria infection status and sex as categorical parametric predictors. *S. mansoni* infection status was included as a binary predictor, with separate models for diagnosis via Kato Katz or POC-CCA ≥ G3. Model selection was carried out in the same way as described above, using AIC and selecting the best fit model.

With all statistical models, the reference group for sex was female and for all binary variables a negative outcome was used as reference.

## Results

### Characterisation of *Schistosoma mansoni* prevalence and intensity of infection in Bugoto, Uganda

Participants were included in the analysis if they had complete data for age, PVD, IP, PSL scores and at least one Kato-Katz sample, totalling 287 participants (Table 1). Most losses from the original target of 400 were due to participants declining an ultrasound or not coming back with parasitological samples. The final sample had an age range of 3–74 years, and a median age of 14, consistent with the median reported in a full community consensus for this area [44]. PSAC represented 15% of the sample, SAC 39% and adults 46%, closely matching the district-wide population structure. However, females were slightly overrepresented in the sample with 63% in comparison to 51% in the district wide census report [50]. This sex difference was observed across all age groups (S1 Table). All participants included in the analysis submitted at least one stool and one urine sample. Three stool samples were collected for 67% (192/287) of all participants, 31% (89/287) provided two samples, and 2% (6/287) provided only one stool sample. Three days of urine samples were collected for 90% of all participants (257/287), 9% (26/287) with samples over two days and (1%) (4/287) with only one urine sample.

**Table 1:** Demographic and infection characteristics of study participants from Bugoto community in May 2022.

Characteristic	n	Prevalence of *S. mansoni* (%)	Arithmetic mean egg count	Geometric mean egg count
**Sex**				
Female	183	57	175	61
Male	104	60	302	132
**Age Group**				
3 - 5	42	43	47	50
6 - 10	72	78	222	131
11 - 14	32	91	276	103
15 - 20	27	78	163	106
21 - 30	30	60	57	27
31 - 40	30	40	32	39
>40	54	24	53	73
**Characteristic**	**n**	**Prevalence in community n = 287 (%)**		
**Intensity of Infection Category (EPG)**				
Negative (0)	120	42		
Light (1–99)	88	31		
Moderate (100–399)	46	16		
Heavy (>400)	33	11		
**Malaria**				
Untested	5	1.7		
Negative	193	67		
Positive	89	31		
**Hookworm**				
Untested	2	0.7		
Negative	246	86		
Positive	39	14		
**Total**	**287**			

*Schistosoma mansoni* prevalence was determined using Kato-Katz (up to six slides) and POC-CCA (G-score ≥3). Arithmetic and geometric mean egg counts (EPG) are reported for infected individuals. Infection intensity was classified as light (1–99 EPG), moderate (100–399 EPG), or heavy (≥400 EPG). Malaria and hookworm status were assessed by rapid diagnostic test and stool microscopy, respectively.

**Fig 1:**
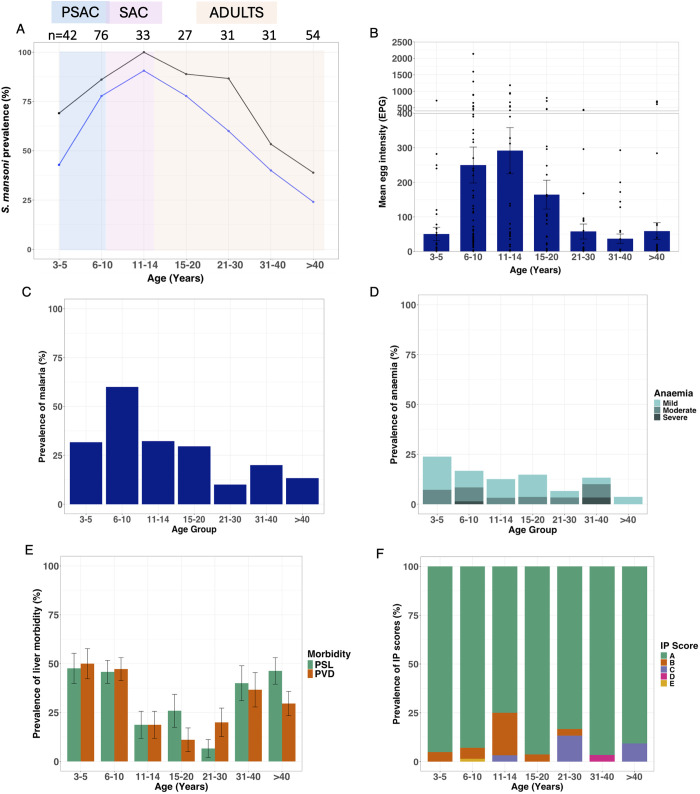
Characterisation of *Schistosoma mansoni* by age in Bugoto, Uganda in 2022. (A) Community prevalence as measured by up to six Kato-Katz slides only (blue line) and Kato-Katz slides plus point-of-care circulating cathode antigen (POC-CCA) G-score ≥3 (black line). Shaded areas are indicating preschool age children (PSAC) = blue, school age children (SAC) = pink and adults = orange. (B) Mean egg intensity as measured by eggs per gram (epg) in faeces, with the separate section at the top indicating those with heavy infections (≥400 epg). (C) Prevalence of malaria; (D) Prevalence of anaemia stratified by mild, moderate and severe. (E) Prevalence of unspecific liver morbidity as measured by portal vein diameter (PVD) and parasternal line of the left liver lobe (PSL). (F). Prevalence of individuals with liver image pattern (IP) scores A-E, and (D) Error bars are standard error of the mean (B), and binary standard errors (E).

Data from the study participants supports the WHO categorisation of Bugoto as being highly endemic for *S. mansoni* with a 65% prevalence in SAC, as measured by just one Kato-Katz slide as per the WHO guidelines. This prevalence increased to 82% when using up to six slides per person and 91% when also including POC-CCA ≥ G3 (Fig 1A - black). When measured for egg positivity by Kato-Katz only (up to six slides), community prevalence was 58% (Fig 1A - blue), however 43 individuals were egg negative by Kato-Katz but positive by POC-CCA. This was disproportionally found in the younger age groups, with 11 samples from the PSAC being negative by Kato-Katz but positive by POC-CCA. Arithmetic mean egg intensity of the whole community was 139 EPG (range: 0–2136) (Fig 1B) and geometric mean of non-zero egg counts was 81 EPG. Both prevalence and intensity of infection peaked in 11–14-year-olds (arithmetic mean = 276, geometric mean = 103) (Table 1). An EPG of ≥400 (heavy infection according to WHO) was found in 11% (32/287) of participants (Table 1 and Fig 1B).

Stool samples examined for non-*S. mansoni* parasite eggs were available for 285 participants. Hookworm infection was identified in 14% (39/285) of the population (Tabe 1), *A. lumbricoides* 0.3%, *H. nana* 0.3%, *E. vermicularis* 0.6% and *T. trichiura* 0.6%. There was no significant difference in prevalence of hookworm by age classes, nor did the likelihood of hookworm infection increase with age. A positive malaria test was observed in 31% (89/282) of the participants. Among age groups, 6–10-year-olds exhibited the highest prevalence of malaria at 60% (42/70) (Fig 1C). Coinfection with malaria, hookworm and *S. mansoni* was observed in 5% of the population, while coinfection of just *S. mansoni* and malaria was more common (26%; 74/282), with the majority of these cases found in the 6–10-year-olds (53%).

The most common self-reported symptoms in all age classes were headache (73%), abdominal pain (64%), diarrhoea (47%) and fever (44%), whilst the lowest reported were body swelling (8%) and difficulty breathing (7%) (Table 2).

**Table 2:** Number and frequency of participants who answered yes to having a self-reported symptom in the last month.

Symptom	PSAC n (%) [Total sample size = 42]	SAC n (%) [Total sample size = 104]	Adults n (%) [Total sample size = 287]	Total n (%) [Total sample size = 287]
Abdominal pain	22 (55)	72 (71)	76 (61)	170 (64)
Blood in stool	6 (15)	30 (29)	16 (13)	52 (20)
Body swelling	2 (5)	9 (9)	10 (8)	21 (8)
Chills	6 (16)	29 (29)	43 (34)	78 (30)
Diarrhoea	25 (63)	52 (53)	46 (37)	123 (47)
Difficulty breathing	2 (5)	5 (5)	11 (9)	18 (7)
Dizziness	6 (15)	43 (42)	63 (51)	112 (42)
Fever	19 (48)	43 (42)	55 (44)	117 (44)
Headache	21 (57)	77 (77)	93 (74)	191 (73)
Muscle pain	4 (10)	10 (10)	48 (38)	62 (23)
Nausea	7 (18)	32 (32)	36 (29)	75 (28)
Pain when urinating	0 (0)	27 (26)	52 (42)	79 (30)
Rash	5 (13)	33 (34)	29 (24)	67 (26)
Vomiting	6 (15)	31 (30)	17 (14)	54 (20)
Weakness	4 (10)	27 (27)	64 (51)	95 (36)

PSAC=preschool age children, SAC=school age children.

Percentage calculated using the total number of individuals in that age group.

Overall, 13% of the population had a positive anaemia diagnosis (n = 287). Anaemia was highest in PSAC, 24% (10/42), followed by 13% in SAC (14/109) and 9% in adults (13/143) (Fig 1D, S1 Table). Of these positive diagnoses more than half were mild anaemia (8% of the participants who tested positive), 5% exhibited moderate anaemia, while notably two individuals presented with severe anaemia (Fig 1D). The first case involved a 38-year-old who tested negative for all parasites screened but showed evidence of enlarged PVD and PSL, this individual reported 12 out of 14 clinical symptoms over the past month, and the sonographer reported gross splenomegaly secondary to portal hypertension. The second case was an 8-year-old who tested negative for malaria, hookworm and other helminths, and was also negative for *S. mansoni* by Kato-Katz but positive with a POC-CCA test. This individual also had PVD and PSL enlargement and reported symptoms of blood in stool, diarrhoea, abdominal pain, and headaches over the past month, both were referred to the local hospital.

A total of 34% (97/287) of participants had a PVD of at least two standard deviations above the Niamey Protocol healthy comparators [8,48]. An increased PSL of the left liver lobe, with a score of at least 2 standard deviations above healthy comparators were found in 33% (94/287) (Fig 1E). No participants were observed to have either collaterals or ascites during ultrasound examination.

Image pattern scores were collected and used to describe a potential for fibrosis, but a positive IP score was not common in this community. Out of the 287 examined by ultrasound, a score of B-F was observed in just 27 participants from the ages of 4–74 years (9%) and 12 with a positive IP score of C-F (4%) (Fig 1E). IP B on its own was highest in SAC (12%, 11/109), and only two cases in each PSAC and adult age groups (Fig 1F). Apart from one 8-year-old with an IP score of E and mean EPG of 2150, all cases of IP scores C and D (n = 11) were observed in the older age groups. These scores were absent from PSAC and younger SAC (6–10-year-olds), with only one case in 11–14-year-olds and most prevalent in the 21–30-year-olds (13%, 4/31) and >40’s (9%, 5/54). An IP score of D, or its combinations, was observed only once in the population and this was in the 31–40-year-old group. Therefore, the trend of more pronounced fibrosis (C-D) appears skewed towards adults. However, the small number of people with any positive IP score in our sample precludes meaningful statistical testing and without the PT score we cannot presume fibrosis. No community member was observed with an IP score of F or combinations which include F. A score which could be deemed as non-schistosomiasis associated (X, Y, Z in the grading system) were found in seven participants (X (cirrhosis)=0, Y (fatty liver)=6 and Z (course liver)=1).

### *Schistosoma mansoni* intensity of infection as a predictor of morbidity

Due to the low number of individuals with a positive IP score, no statistical analysis could be conducted for this morbidity marker, as the sample size was insufficient to provide reliable estimates or ensure statistical power for meaningful comparisons. Out of the STHs measured, only hookworm was included in the statistical modelling as all other had a prevalence <1%.

**Fig 2:**
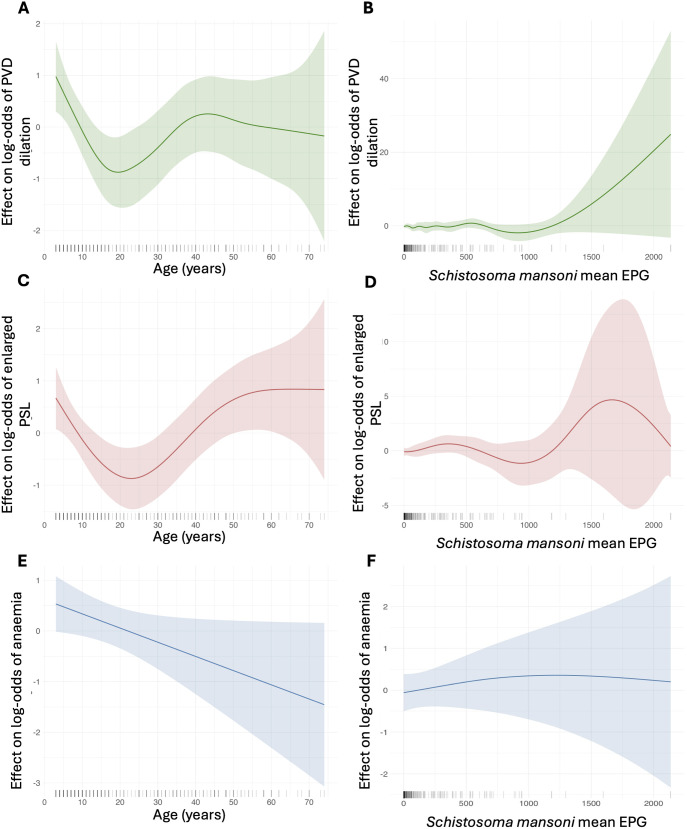
Effect plots from generalised additive models (GAMs) showing the smooth effects of age (left column) and mean *Schistosoma mansoni* infection intensity (right column) on the log-odds of three morbidity outcomes: portal vein diameter dilation (PVD [green]), enlarged parasternal line (PSL [red]), and anaemia (blue). Shaded ribbons represent 95% confidence intervals around the fitted smooth curves. Tick marks along the x-axis indicate the distribution of observations. Broader confidence intervals at higher EPG reflect lower sample sizes.

When using PVD as the response variable, the best-fitting GAM model included no interactions, used cubic regression splines, the REML method for smoothing parameter estimation of age and smoothing parameter = 0.1 for *S. mansoni i*nfection intensity. The model indicated that *S. mansoni* infection intensity was not significantly associated with the likelihood of PVD dilation (edf = 8.24, χ² = 8.57, p = 0.796) (Table 3). Age was the most significant predictor of PVD dilation (edf = 4.08, χ² = 14.38, p = 0.014). Malaria infection was also positively associated with PVD dilation (est = 0.70, z = 2.25, p = 0.025) as was sex, with males more likely to test positive for PVD than females (est = 0.60, z = 2.07, p = 0.039) (Table 3). Hookworm infection was not significantly associated with the likelihood of PVD. The predicted probability of PVD was highest at the youngest observed age (3 years), declines to a minimum of around 20 years of age, then gradually increases again, plateauing around age 43 (Fig 2A). Additionally, the effect of *S. mansoni* intensity on PVD is held constant with no effect until approximately 1200 EPG, after which there is a steep rise in the likelihood of PVD with increasing EPG observed (Fig 2B). However, with very wide confidence intervals due to low sample sizes in these high egg counts inferences concerning *S. mansoni* intensity at high EPG and PVD dilation should be made with caution.

**Table 3:** GAM model summaries: *Schistosoma mansoni* mean intensity as a predictor for portal vein dilation (PVD), enlarged parasternal line (PSL) and anaemia.

	PVD			
	Parametric coefficients			
*Term*	*estimate*	*std. error*	*z-value*	*p.value*
Intercept	-1.07	0.24	-4.54	<0.001
Hookworm	0.51	0.41	1.25	0.211
Malaria	0.70	0.31	2.25	**0.025**
Sex	0.60	0.29	2.07	**0.039**
	**Smooth terms**			
	*edf*	*Ref.df*	*Chi.sq*	*p-value*
*S. mansoni* mean intensity	8.24	8.74	5.57	0.796
Age	4.08	5.02	14.38	**0.014**
	**PSL**			
	**Parametric coefficients**			
*Term*	*estimate*	*std. error*	*z-value*	*p.value*
Intercept	-0.52	0.20	-2.67	0.008
Hookworm	-0.40	0.41	-0.98	0.327
Malaria	0.58	0.30	1.93	0.054
Sex	-0.56	0.29	-1.97	**0.049**
	**Smooth terms**			
	*edf*	*Ref.df*	*Chi.sq*	*p-value*
*S. mansoni* mean intensity	5.70	6.36	5.06	0.583
Age	3.37	4.06	16.46	**0.003**
	**Anaemia**			
	**Parametric coefficients**			
*Term*	*estimate*	*std. error*	*z-value*	*p.value*
Intercept	-2.35	0.30	-7.97	<0.001
Hookworm	-0.10	0.53	-0.19	0.847
Malaria	0.72	0.38	1.92	0.056
Sex	0.30	0.37	0.82	0.413
	**Smooth terms**			
	*edf*	*Ref.df*	*Chi.sq*	*p-value*
*S. mansoni* mean intensity	1.00	1.00	0.28	0.594
Age	1.00	1.00	2.96	0.085

PVD = portal vein dilation, PSL = parasternal line, POC-CCA = Point-of-care circulating cathodic antigen, std = standard, edf = estimated degrees of freedom, Ref.df = reference degrees of freedom.

Considering PSL, the best-fitting GAM model did not include any interactions, used P-splines, the REML method for smoothing estimation of the age parameter and smoothing parameter = 0.1 for mean intensity of *S. mansoni* infection. The model indicated that *S. mansoni* infection intensity was not significantly associated with the likelihood of PSL (edf = 5.70, χ² = 5.06, p = 0.583) (Table 3). Like the PVD model, age was the main significant predictor of enlarged PSL (edf = 3.37, χ² = 16.46, p = 0.003) and sex showed a marginally significant negative effect, indicating that females presented with slightly more PSL than males (est = -0.56, z = -1.97, p = 0.049) (Table 3). There was a positive, marginally significant trend in the relationship between malaria positivity and PSL (est = 0.58, z = 1.93, p = 0.054). Hookworm infection was not significantly associated with PSL enlargement (Table 2). Predicted effect plots of age against the effect on PSL showed that the probability of PSL was high in the three year olds, declined to a minimum at approximately 23 years of age, then gradually increased again to just above the level of the three year olds, plateauing around age 55 (Fig 2C). The predicted effect plot of *S. mansoni* intensity against effect of PSL confirmed that there was no effect of *S. mansoni* intensity on the probability of PSL enlargement. An exception was observed at approximately 1800 EPG, where the probability increased before subsequently decreasing again (Fig 2D).

To investigate the relationship between anaemia and *S. mansoni* infection intensity the best-fitting GAM model had no interactions, used cubic regression splines, and a smoothing parameter of 0.2. There was no significant association between anaemia and *S. mansoni* mean intensity of infection, nor a current hookworm infection (Table 3). Being positive for malaria had a weak positive trend with anaemia (est = 0.72, z-value = 1.92, p = 0.056) (Table 3). Age and intensity of infection both had non-significant negative linear effects with anaemia probability, with the effect of age being most pronounced (edf = 1.00, χ² = 2.96, p = 0.085) (Table 3). The predictive plots of smooth estimates showed the highest probability of anaemia was observed in the younger age groups, which then declines with age (Fig 2E). Mean intensity of *S. mansoni* had no observable effect of anaemia likelihood (Fig 2F).

Regarding the associations between *S. mansoni* infection intensity, age, co-infections and fifteen separate self-reported symptoms (S5 Table), across all models *S. mansoni* intensity was not significantly associated with any of the binary symptom outcomes, there was a non-significant trend towards a linear association between *S. mansoni* intensity and self-reported weakness (edf = 1.00, χ² = 2.80, p = 0.094). Age emerged as the most consistent predictor showing a statistically significant non-linear relationship with 7 out of the 15 morbidity symptoms: abdominal pain (edf = 3.66, χ² = 10.96, p = 0.041), chills (edf = 4.30, χ² = 5.33, p = 0.003), dizziness (edf = 2.92, χ² = 11.53, p = 0.016), nausea (edf = 2.63, χ² = 9.29, p = 0.033), pain when urinating (edf = 4.53, χ² = 30.46, p < 0.001), rash (edf = 6.61, χ² = 23.14, p = 0.005), and weakness (edf = 3.31, χ² = 24.10, p < 0.001) S3 Table. Hookworm and malaria infections also showed non-significant positive associations with symptoms when controlling for other covariates, although not significant a negative association between diarrhoea and hookworm (est = -0.89, z = -1.90, p = 0.058), and a positive association between nausea and malaria (est = 0.67, z = 1.87, p = 0.061) was observed (S3 Table).

### *Schistosoma mansoni* infection (measured by Kato Katz or POC-CCA) as a predictor of morbidity

When using *S. mansoni* infection status (positive vs. negative, based on Kato-Katz) along with age, malaria, and hookworm as predictors, model fit for the anaemia model was best without including sex, and it was therefore excluded from the model. In contrast, sex was retained as a covariate in the PVD and PSL models. Across all models, each with a different morbidity marker as the response (PVD dilation, PSL enlargement, or anaemia), *S. mansoni* infection was not significantly associated with increased morbidity (S4 Table). Age was a significant predictor in all models. Malaria was significantly associated with anaemia (estimate = 0.79, z = 2.08, p = 0.038), and showed weak positive trends for PVD (estimate = 0.50, z = 1.63, p = 0.10) and PSL (estimate = 0.50, z = 1.70, p = 0.089), though these were not statistically significant (S4 Table).

When the same models were run using POC-CCA ≥ G3 as the diagnostic method for *S. mansoni*, there were no significant differences in model outcomes to the Kato-Katz models (S5 Table). Being positive for *S. mansoni* based on POC-CCA ≥ G3 was not associated with PVD dilation, PSL enlargement, or anaemia. Malaria continued to show a positive trend with PVD, PSL and anaemia (S5 Table).

When using the fifteen self-reported symptoms as the binary response variables, we observed a similar trend with only age being a significant predictor of symptoms (S6 Table). However, when we used POC-CCA ≥ G3 as a predictor instead of Kato Katz, along with age, we observed a significant association with blood in stool (est = 1.59, z = 2.29, p = 0.022) and a positive trend between itchy rash and infection diagnosed by POC-CCA (est = 0.86, z = 1.81, p = 0.071) (S5 Table). In both models (Kato Katz and POC-CCA), although not significant, malaria tended to be positively associated with nausea and vomiting and had a negative association with muscle pain (S6 and S7 Tables).

## Discussion

Here we observed extremely high *S. mansoni* prevalence levels in Bugoto, Uganda, with 100% of 11–14-year-olds and 73% of the overall population infected with *S. mansoni* (based on Kato-Katz and/or POC-CCA diagnostics). We found relatively low to moderate infection intensities on average (arithmetic mean = 139 EPG, geometric mean = 81 EPG) and low prevalence of image pattern scores which could indicate liver fibrosis (IP(B-F) = 9%; IP(C-F) = 4%). However, counter to our expectations, we observed a moderate prevalence of other morbidity markers including PVD (34%), PSL (33%) and anaemia (13%) in this community.

Contrary to our initial hypothesis that morbidity would be highest in adults due to cumulative infections, PSAC showed the highest burdens: 50% had PVD dilation, 48% had enlarged PSL, and 24% were anaemic. This could be explained by immunological differences in developing immune systems. PSAC may produce a less effective Th-2-mediated granulomatous response, leading to reduced egg excretion and increased tissue egg retention, exacerbating morbidity even in low-intensity infections [51,52]. Supporting this, 43 individuals were egg-negative by Kato-Katz but positive by POC-CCA, with younger children overrepresented suggesting either undetected low-intensity infections or increased tissue egg deposition. Moreover, a limitation of our study was the absence of individual-level data on treatment history or duration of residence, making it uncertain whether adults have had continuous exposure. In contrast, PSAC are more likely to have lived their entire lives by the lake and to be treatment naive. Therefore, these factors may explain the unexpectedly high morbidity observed in PSAC and highlight the importance of using sensitive diagnostics such as POC-CCA, particularly in younger children, where low-intensity or tissue-localised infections may go undetected.

A recent study of PSAC in another highly schistosomiasis-endemic region of Uganda observed similar results: where 8% of the PSAC living in communities near Lake Albert had periportal fibrosis (IP (B-F score); [53]), comparable to the 5% we observed in Bugoto PSAC. However, we found much higher levels of other morbidity markers (PVD and PSL) in contrast to Lake Albert community PSAC who had no apparent signs of PVD and only 0.6% had enlarged PSL. These differences may be explained by methodological and epidemiological factors. While Pach *et al.* used a stricter ≥4 SD threshold to define morbidity, we applied a more sensitive ≥2 SD cut-off. Applying their threshold to our data reduces PVD prevalence to 0%, but PSL remains at 7%. Higher malaria prevalence (32% vs. 16%) may contribute to this difference as malaria was positively associated with PSL in our cohort, and higher previous exposure would likely be higher in our study location. The similar IP scores observed in both our study and Pach *et al*. (2024) suggest that early-life exposure to *S. mansoni* is sufficiently intense to initiate liver pathology regardless of region and, despite differences, both studies indicate the presence of incipient morbidity resulting from early infections and add further evidence to the need for exploration of the patterns of morbidity and morbidity risk factors among PSAC in diverse geographical regions.

An image pattern of B when measuring periportal fibrosis is often not measured due to its indeterminate nature [8], and although we cannot comment on fibrosis due to not having a corresponding PT score (see limitations below), we observed an image score of B almost exclusively in the 3–14-year-olds. A similar study which took place in Uganda from 2003-2005 also found only cases of periportal fibrosis with an image pattern of B in SAC, which they suggested could indicate early changes which, without treatment, would have progressed to more severe fibrosis [41]. The fact that these changes were no longer detectable after treatment in this study further supports the interpretation that IP score B may represent early liver changes directly attributable to *S. mansoni* infection. This finding almost 20 years prior to our own study highlights that this is not a new phenomenon but underscores that IP score B is a consistent marker of early morbidity. With the relative success of two decades of MDAs, particularly in SAC, potentially resulting in the lower incidence of severe morbidities traditionally found in older age groups (as were also found in our population, albeit at extremely low levels), the focus of studies are changing and looking at these more subtle clinical manifestations such as IP score B, which could characterise emerging morbidity in younger age groups.

The age associated distribution we observed could be linked to the timing of MDAs and the impact of sustained treatment in the face of rapid reinfection over many years. This study took place in May 2022 prior to any MDA treatments that year. In Bugoto, annual MDA of SAC began in 2003 [25,41], extending to the whole community (excluding PSAC) from 2004 and became biannual in 2019 [38]. Consequently, PSAC within this study cohort would not have been part of MDA. SAC would have experienced between 1 and 12 MDAs based on their age, and adults could have undergone a maximum of 22 MDAs throughout their lifetime if they had lived in this region since the control programme started. However, MDA coverage in Bugoto has been very low, especially in adults [44]. Furthermore, individuals born before the initiation of nationwide praziquantel MDA programmes would have experienced a longer period of untreated exposure during early life. This may contribute to the lower *S. mansoni*-specific IP scores observed in 15–20-year-olds, who likely benefited from regular MDA during childhood. Our results using PVD and PSL support this interpretation, showing a loss of the protective effect of treatment in adults over 40 years old, a group who were already aged 20 or more when praziquantel MDA began and may have sustained irreversible hepatic damage before treatment was available. Importantly, these findings strengthen the evidence that praziquantel may reduce schistosomiasis-associated morbidity, while also highlighting the need for early intervention. Starting treatment at school age may come too late for some children, as shown by the presence of extensive fibrosis in an 8-year-old and the high prevalence of PVD, PSL, and anaemia in PSAC in our study.

Importantly, we found no clear association between most of our morbidity markers and current *S. mansoni* infections. Only self-reported blood in stool was associated with current infection when measured with POC-CCA. The observed association between self-reported blood in stool and POC-CCA positivity, but not with Kato-Katz results, could be due to blood in stool not exclusively being a result of high-intensity infections, but rather from factors such as mucosal inflammation or immune-mediated tissue damage, which can occur even in lower-intensity infections. Because POC-CCA detects circulating antigens from live adult worms regardless of egg output, it may identify individuals with ongoing worm activity and associated morbidity that Kato-Katz misses, particularly in those with intermittent or low egg shedding and subtle morbidity.

According to the WHO thresholds, the Ugandan community examined here does not meet the criteria for having achieved either morbidity control (<5% heavy infections) or EPHP (<1% heavy infections) [15], as 11% of the population had heavy infections. Furthermore, morbidity was evident even among those individuals with light or moderate infections. While we did observe a trend of increasing PVD and PSL at very high EPG (>1200 and 1800 respectively), this represented a very small number of individuals and lies well beyond the WHOs threshold for heavy infection (≥400 EPG). This raises questions about the adequacy of these thresholds in reflecting actual morbidity risk and important ethical considerations regarding subtle morbidities and whether these are a health burden to individuals, their families and communities [45].

A current malaria infection was common amongst the participants (31%), being most prevalent in the younger age groups. It was associated with PVD, PSL and anaemia, mirroring the age-related patterns of morbidity, therefore indicating that either undetectable *S. mansoni* infection or a malaria infection, or a combination could be driving our results. Researchers observed a significant relationship between PSL and both *S. mansoni* infection and malaria infection in PSAC in Rusinga island, Kenya (also in the north eastern area of Lake Victoria) [10]. As the prevalence of PSL did not decrease after praziquantel treatment they associated this with an increase in malaria prevalence during the study. However, treatment with praziquantel does not always reduce S*chistosoma*-associated morbidity [26,28,54], therefore the observed hepatomegaly may not have been exclusively caused by malaria infection in Rusinga island. When examining the role of a hookworm infection and morbidity in our study, there was no association between a detected hookworm infection and any of the morbidity markers in our study group. This contrasts with previous findings in the same district in 2013 which found a significant association between anaemia and having a heavy hookworm infection. These differences are likely due to the much higher prevalence of hookworm and anaemia at that earlier time-point, which were 41% and 44% respectively, rather than 14% and 13% observed in our study, and perhaps an indication of successful STH treatment programs which have run alongside *Schistosoma* MDAs since 2003. These studies along with our own findings highlight the difficulty in disentangling the aetiology of morbidities in regions with co-endemicity and the importance of integrated control programs which have overall benefits in reducing parasite burden and shared associated morbidity.

As we contemplate the new guideline of biannual treatment in all regions with over 50% prevalence [32], we anticipate a decline in severe morbidities [36]. However, this shift will bring new challenges in detecting and managing subtle morbidities, therefore underscoring the need for comprehensive approaches that safeguard the health and well-being of affected communities and individuals of all ages.

## Limitations

A measurement of the second order branches used to establish a PT score according to the Niamey protocol were not measured during this study due to constraints with the equipment available. As periportal fibrosis is estimated using a combination of both IP and PT score, we could not accurately report on fibrosis in this study, therefore we gave a description of the IP scores without indicating fibrosis.

Generalisability of our findings is limited by the fact that results are based on a single cross-sectional study in one community in Mayuge District, so our results should be used to complement longitudinal, population-level analyses by capturing under-documented morbidity patterns across all age groups. Additionally, our sample included a higher proportion of female participants (63%), higher than the district average of 51%. This sex imbalance may have influenced the morbidity patterns we observed. For instance, adult males, who exhibited a higher intensity of infection than females, were underrepresented, potentially leading to an underestimation of chronic morbidity in the adult population.

Although the Niamey protocol provides organometric measurements for comparison of height matched individuals with PVD and PSL scores, the recommendation was always to use values from healthy people of the same ethnic group and region [8]. However, in practice this is rarely undertaken, and the Senegalese controls provided in the protocol are usually used, for example [10,27,40,53,55–57] often due to difficulties in finding truly uninfected and unexposed controls from these highly endemic regions. We explored whether those with undetectable *S. mansoni* infection in our study community had higher morbidity than those in the Sudanese population and found consistently higher measurements for PSL and PVD in the participants from Bugoto (S1 Fig). This could indicate that previous infections leave lasting damage or that subclinical and sub-diagnostic infections, or parasite exposures are indeed causing harm. However, these differences could also be due to inter-site variation [30] or due to the lack of malaria in this Senegal region. This reliance on external comparator data could have introduced misclassification in our designation of morbidity, potentially overestimating the prevalence of enlarged PSL or dilated PVD if the Senegalese reference values underestimate normal liver dimensions for our Ugandan population. Conversely, if underlying morbidity exists even in individuals who test negative for *S. mansoni*, then our approach may have helped reveal true but subtle disease that would otherwise go unrecognised.

## Supporting information

S1 Fig Liver morbidity in *Schistosoma mansoni* uninfected individuals from Bugoto compared with comparators data from *S. mansoni* non-endemic areas. The colour PSL ranges are based on the Naimey protocol: normal range (green), moderate abnormal (yellow) and abnormal (red) measurements for (A) portal vein dilation and (B) left parasternal line. Purple line is the simulated data using 1000 bootstraps with sample size matching for height category with the Bugoto study data, and the mean and standard deviation from the Niamey protocol.
(TIF)

S1 Table Arithmetic and geometric mean EPG across age and sex classes. (DOCX)

S2 Table Frequency of anaemia by age group. (DOCX)

S3 Table GAM model summaries: Schistosoma mansoni mean intensity as a predictor for self-reported symptoms. (DOCX)

S4 Table GAM model summaries: *Schistosoma mansoni* infection as measured by Kato-Katz as a predictor for portal vein dilation (PVD), enlarged parasternal line (PSL) and anaemia. (DOCX)

S5 Table GAM model summaries: *Schistosoma mansoni* infection as measured by POC-CCA as a predictor for portal vein dilation (PVD), enlarged parasternal line (PSL) and anaemia. (DOCX)

S6 Table GAM model summaries: Schistosoma mansoni infection (Kato Katz) as a predictor for self-reported symptoms. (DOCX)

S7 Table GAM model summaries: Schistosoma mansoni infection (POC-CCA) as a predictor for self-reported symptoms. (DOCX)
