# Supplementary figures and images for: The relationship between expelled eggs, morbidity and age in a Schistosoma mansoni endemic setting in Uganda: Implications for current elimination policies
Source: PLoS Negl Trop Dis. 2025 Sep 3;19(9):e0012750. doi: 10.1371/journal.pntd.0012750 (PMC12407471; doi:10.1371/journal.pntd.0012750)

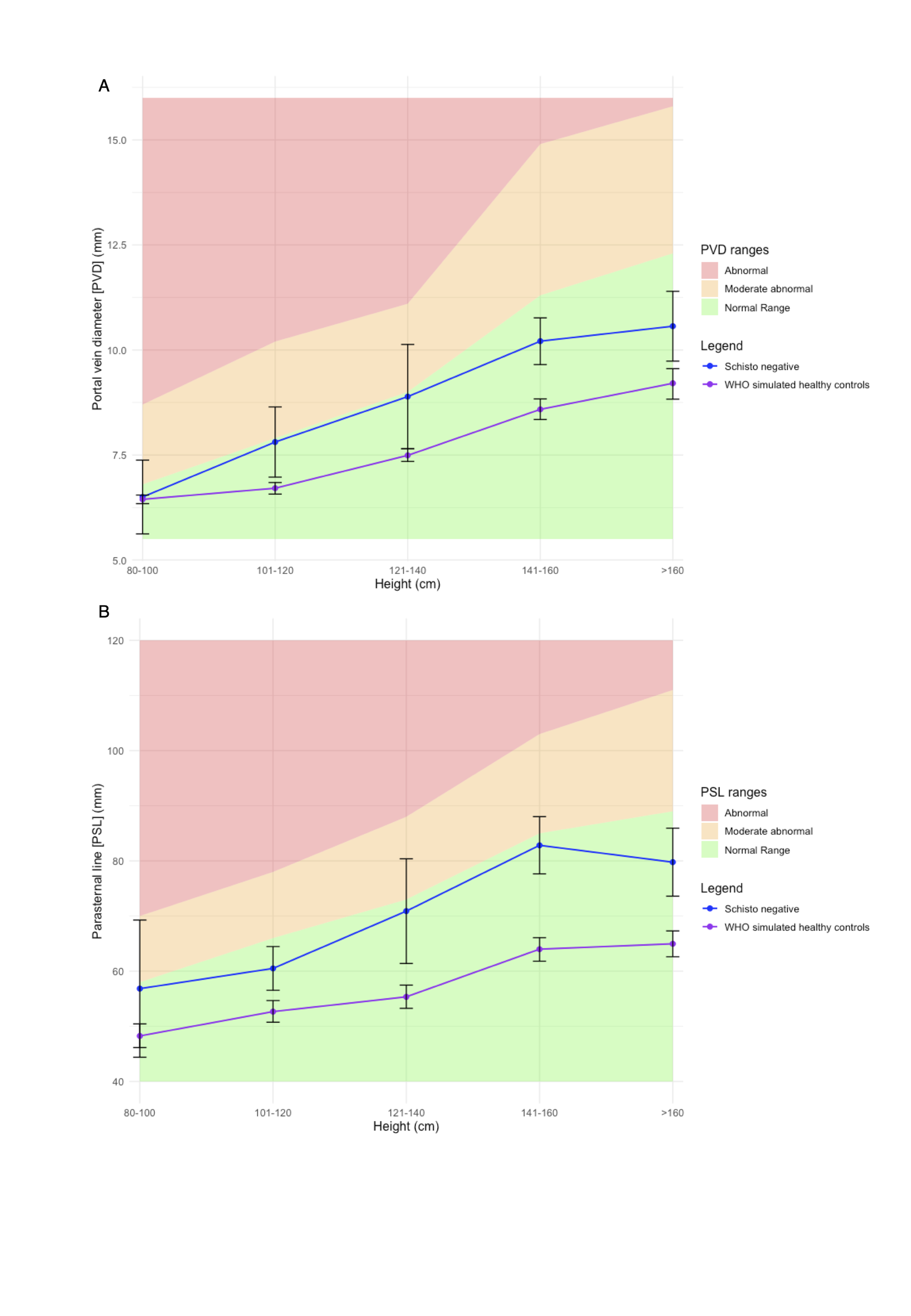

Supplement: S1 Fig — The colour PSL ranges are based on the Naimey protocol: normal range (green), moderate abnormal (yellow) and abnormal (red) measurements for (A) portal vein dilation and (B) left parasternal line. Purple line is the simulated data using 1000 bootstraps with sample size matching for height category with the Bugoto study data, and the mean and standard deviation from the Niamey protocol. (TIF) [file pntd.0012750.s001.tif]
