# Supplementary material for: The relationship between expelled eggs, morbidity and age in a Schistosoma mansoni endemic setting in Uganda: Implications for current elimination policies
Source: PLoS Negl Trop Dis. 2025 Sep 3;19(9):e0012750. doi: 10.1371/journal.pntd.0012750 (PMC12407471; doi:10.1371/journal.pntd.0012750)
Supplement: S1 Table — (DOCX) [file pntd.0012750.s002.docx]

| ***S1 Table. Arithmetic and geometric mean EPG across age and sex classes*** | | | | |
| --- | --- | --- | --- | --- |
| **Age class** | **Sex** | **n** | **Arirthmic mean EPG** | **Geometric mean EPG** |
| PSAC | F | 26 | 71.4 | 49.6 |
| PSAC | M | 16 | 16.5 | 50.8 |
| SAC | F | 65 | 212 | 92.7 |
| SAC | M | 48 | 306 | 152 |
| Adults | F | 92 | 55.9 | 38.6 |
| Adults | M | 40 | 105 | 118 |
|  |  |  |  |  |

*EPG=eggs per gram, PSAC= preschool age children, SAC = school age children, F=female, M=male*
