# Supplementary material for: The relationship between expelled eggs, morbidity and age in a Schistosoma mansoni endemic setting in Uganda: Implications for current elimination policies
Source: PLoS Negl Trop Dis. 2025 Sep 3;19(9):e0012750. doi: 10.1371/journal.pntd.0012750 (PMC12407471; doi:10.1371/journal.pntd.0012750)
Supplement: S2 Table — (DOCX) [file pntd.0012750.s003.docx]

***S2 Table. Frequency of anaemia by age group.***

| *Age group* | *Percent positive for anaemia (%)* | *Age class* | *Percent positive for anaemia (%)* |
| --- | --- | --- | --- |
| *3-5* | *24.4* | *PSAC* | *24.4* |
| *6-10* | *15.7* | *SAC* | *13.6* |
| *11-14* | *12.9* |  |  |
| *15-20* | *14.8* | *Adults* | *9.2* |
| *21-30* | *6.7* |  |  |
| *31-40* | *13.3* |  |  |
| *>40* | *3.8* |  |  |
