# Supplementary material for: The relationship between expelled eggs, morbidity and age in a Schistosoma mansoni endemic setting in Uganda: Implications for current elimination policies
Source: PLoS Negl Trop Dis. 2025 Sep 3;19(9):e0012750. doi: 10.1371/journal.pntd.0012750 (PMC12407471; doi:10.1371/journal.pntd.0012750)
Supplement: S3 Table — (DOCX) [file pntd.0012750.s004.docx]

| **S3 Table. GAM model summaries: *Schistosoma mansoni* mean intensity as a predictor for self-reported symptoms** | | | | |  |  |  |  |  |  |
| --- | --- | --- | --- | --- | --- | --- | --- | --- | --- | --- |
| **Symptom** | **Type** | **Term** | **Estimate** | **Std.Error** | | **z-value** | **p.value** | **edf** | **Ref.df** | **Chi.sq** |
| Abdominal Pain | Parametric | Intercept | 0.560 | 0.190 | | 2.970 | 0.003 |  |  |  |
| Abdominal Pain | Parametric | Hookworm | -0.580 | 0.420 | | -1.370 | 0.170 |  |  |  |
| Abdominal Pain | Parametric | Malaria | -0.030 | 0.340 | | -0.090 | 0.928 |  |  |  |
| Abdominal Pain | Smooth | *S. mansoni* mean intensity |  |  | |  | 0.175 | 1.000 | 1.000 | 1.840 |
| Abdominal Pain | Smooth | Age |  |  | |  | 0.041 | 3.660 | 4.530 | 10.960 |
| Blood in stool | Parametric | Intercept | -4.210 | 6.330 | | -0.670 | 0.505 |  |  |  |
| Blood in stool | Parametric | Hookworm | -0.870 | 0.700 | | -1.240 | 0.214 |  |  |  |
| Blood in stool | Parametric | Malaria | 0.280 | 0.450 | | 0.620 | 0.534 |  |  |  |
| Blood in stool | Smooth | *S. mansoni* mean intensity |  |  | |  | 0.947 | 4.940 | 5.530 | 1.500 |
| Blood in stool | Smooth | Age |  |  | |  | 0.381 | 11.490 | 12.990 | 13.820 |
| Body swelling | Parametric | Intercept | -2.620 | 0.360 | | -7.320 | 0.000 |  |  |  |
| Body swelling | Parametric | Hookworm | -0.720 | 1.27E+07 | | 0.000 | 1.000 |  |  |  |
| Body swelling | Parametric | Malaria | 0.390 | 0.620 | | 0.630 | 0.528 |  |  |  |
| Body swelling | Smooth | *S. mansoni* mean intensity |  |  | |  | 0.822 | 1.000 | 1.000 | 0.050 |
| Body swelling | Smooth | Age |  |  | |  | 0.781 | 1.420 | 1.730 | 0.290 |
| Chills | Parametric | Intercept | -1.200 | 0.230 | | -5.170 | 0.000 |  |  |  |
| Chills | Parametric | Hookworm | -0.200 | 0.490 | | -0.420 | 0.677 |  |  |  |
| Chills | Parametric | Malaria | 0.170 | 0.380 | | 0.440 | 0.660 |  |  |  |
| Chills | Smooth | *S. mansoni* mean intensity |  |  | |  | 0.511 | 1.000 | 1.000 | 0.430 |
| Chills | Smooth | Age |  |  | |  | 0.003 | 4.300 | 5.330 | 18.480 |
| Diarrhoea | Parametric | Intercept | -0.170 | 0.190 | | -0.890 | 0.374 |  |  |  |
| Diarrhoea | Parametric | Hookworm | -0.890 | 0.470 | | -1.900 | 0.058 |  |  |  |
| Diarrhoea | Parametric | Malaria | -0.020 | 0.350 | | -0.050 | 0.958 |  |  |  |
| Diarrhoea | Smooth | *S. mansoni* mean intensity |  |  | |  | 0.716 | 6.370 | 7.120 | 5.070 |
| Diarrhoea | Smooth | Age |  |  | |  | 0.147 | 7.020 | 7.730 | 12.220 |
| Difficulty breathing | Parametric | Intercept | -4.460 | 1.440 | | -3089.000 | 0.002 |  |  |  |
| Difficulty breathing | Parametric | Hookworm | -14.950 | 738.220 | | -0.020 | 0.984 |  |  |  |
| Difficulty breathing | Parametric | Malaria | 0.200 | 0.820 | | 0.240 | 0.813 |  |  |  |
| Difficulty breathing | Smooth | *S. mansoni* mean intensity |  |  | |  | 0.200 | 3.870 | 4.590 | 6.180 |
| Difficulty breathing | Smooth | Age |  |  | |  | 0.724 | 4.500 | 5.530 | 3.320 |
| Dizziness | Parametric | Intercept | -0.360 | 0.190 | | -1.930 | 0.054 |  |  |  |
| Dizziness | Parametric | Hookworm | 0.260 | 0.430 | | 0.600 | 0.550 |  |  |  |
| Dizziness | Parametric | Malaria | -0.460 | 0.350 | | -1.320 | 0.188 |  |  |  |
| Dizziness | Smooth | *S. mansoni* mean intensity |  |  | |  | 0.865 | 1.000 | 1.000 | 0.030 |
| Dizziness | Smooth | Age |  |  | |  | 0.016 | 2.920 | 3.650 | 11.530 |
| Fever | Parametric | Intercept | -0.170 | 0.180 | | -0.950 | 0.340 |  |  |  |
| Fever | Parametric | Hookworm | -0.260 | 0.420 | | -0.620 | 0.535 |  |  |  |
| Fever | Parametric | Malaria | -0.030 | 0.320 | | -0.100 | 0.918 |  |  |  |
| Fever | Smooth | *S. mansoni* mean intensity |  |  | |  | 0.678 | 1.000 | 1.000 | 0.170 |
| Fever | Smooth | Age |  |  | |  | 0.202 | 1.000 | 1.000 | 1.630 |
| Headache | Parametric | Intercept | 0.970 | 0.200 | | 4.950 | 0.000 |  |  |  |
| Headache | Parametric | Hookworm | -0.600 | 0.420 | | -1.410 | 0.158 |  |  |  |
| Headache | Parametric | Malaria | 0.210 | 0.360 | | 0.590 | 0.558 |  |  |  |
| Headache | Smooth | *S. mansoni* mean intensity |  |  | |  | 0.870 | 1.130 | 1.240 | 0.180 |
| Headache | Smooth | Age |  |  | |  | 0.420 | 1.190 | 1.360 | 0.770 |
| Muscle Pain | Parametric | Intercept | -2.180 | 0.590 | | -3.680 | 0.000 |  |  |  |
| Muscle Pain | Parametric | Hookworm | 0.350 | 0.590 | | 0.600 | 0.550 |  |  |  |
| Muscle Pain | Parametric | Malaria | -0.790 | 0.590 | | -1.340 | 0.180 |  |  |  |
| Muscle Pain | Smooth | *S. mansoni* mean intensity |  |  | |  | 0.429 | 7.110 | 7.740 | 7.780 |
| Muscle Pain | Smooth | Age |  |  | |  | 0.284 | 8.680 | 8.970 | 10.730 |
| Nausea | Parametric | Intercept | -1.190 | 0.220 | | -5.520 | 0.000 |  |  |  |
| Nausea | Parametric | Hookworm | 0.120 | 0.450 | | 0.260 | 0.793 |  |  |  |
| Nausea | Parametric | Malaria | 0.670 | 0.360 | | 1.870 | 0.061 |  |  |  |
| Nausea | Smooth | *S. mansoni* mean intensity |  |  | |  | 0.216 | 1.000 | 1.000 | 1.530 |
| Nausea | Smooth | Age |  |  | |  | 0.033 | 2.630 | 3.220 | 9.290 |
| Pain when Urinating | Parametric | Intercept | -0.970 | 0.250 | | -3.920 | 0.000 |  |  |  |
| Pain when Urinating | Parametric | Hookworm | -0.460 | 0.510 | | -0.900 | 0.370 |  |  |  |
| Pain when Urinating | Parametric | Malaria | -0.400 | 0.420 | | -0.960 | 0.339 |  |  |  |
| Pain when Urinating | Smooth | *S. mansoni* mean intensity |  |  | |  | 0.628 | 3.210 | 3.850 | 2.450 |
| Pain when Urinating | Smooth | Age |  |  | |  | 0.000 | 4.580 | 5.360 | 30.460 |
| Rash | Parametric | Intercept | -1.260 | 0.230 | | -5605.000 | 0.000 |  |  |  |
| Rash | Parametric | Hookworm | -0.110 | 0.500 | | -0.230 | 0.818 |  |  |  |
| Rash | Parametric | Malaria | -0.130 | 0.390 | | -0.340 | 0.738 |  |  |  |
| Rash | Smooth | *S. mansoni* mean intensity |  |  | |  | 0.358 | 1.000 | 1.000 | 0.840 |
| Rash | Smooth | Age |  |  | |  | 0.005 | 6.610 | 7.660 | 23.140 |
| Vomiting | Parametric | Intercept | -1.600 | 0.250 | | -6.430 | 0.000 |  |  |  |
| Vomiting | Parametric | Hookworm | -0.060 | 0.520 | | -0.130 | 0.900 |  |  |  |
| Vomiting | Parametric | Malaria | 0.580 | 0.380 | | 1.520 | 0.128 |  |  |  |
| Vomiting | Smooth | *S. mansoni* mean intensity |  |  | |  | 0.352 | 2.510 | 3.100 | 3.380 |
| Vomiting | Smooth | Age |  |  | |  | 0.199 | 2.380 | 2.980 | 4.890 |
| Weakness | Parametric | Intercept | -0.800 | 0.220 | | -3.720 | 0.000 |  |  |  |
| Weakness | Parametric | Hookworm | -0.800 | 0.520 | | -1.540 | 0.123 |  |  |  |
| Weakness | Parametric | Malaria | -0.310 | 0.400 | | -0.770 | 0.439 |  |  |  |
| Weakness | Smooth | *S. mansoni* mean intensity |  |  | |  | 0.094 | 1.000 | 1.000 | 2.800 |
| Weakness | Smooth | Age |  |  | |  | 0.000 | 3.310 | 4.110 | 24.100 |

*PVD=portal vein dilation, PSL=parasternal line, KK= Kato-Katz, std=standard, edf=estimated degrees of freedom, Ref.df=reference degrees of freedom.*
