# Supplementary material for: The relationship between expelled eggs, morbidity and age in a Schistosoma mansoni endemic setting in Uganda: Implications for current elimination policies
Source: PLoS Negl Trop Dis. 2025 Sep 3;19(9):e0012750. doi: 10.1371/journal.pntd.0012750 (PMC12407471; doi:10.1371/journal.pntd.0012750)
Supplement: S4 Table — (DOCX) [file pntd.0012750.s005.docx]

| **S4 Table. GAM model summaries: *Schistosoma mansoni* infection as measured by Kato-Katz as a predictor for portal vein dilation (PVD), enlarged parasternal line (PSL) and anaemia** | | | | |
| --- | --- | --- | --- | --- |
|  | **PVD** | | | |
|  | **Parametric coefficients** | | | |
| *Term* | *estimate* | *std. error* | *statistic* | *p.value* |
| Intercept | -1.17 | 0.27 | -4.28 | <0.001 |
| Hookworm | 0.41 | 0.40 | 1.02 | 0.307 |
| Malaria | 0.50 | 0.30 | 1.63 | 0.103 |
| Sex | 0.51 | 0.28 | 1.80 | 0.072 |
| *S. mansoni* -KK | 0.02 | 0.31 | 0.07 | 0.941 |
|  | **Smooth terms** | | | |
|  | *edf* | *Ref.df* | *Chi.sq* | *p-value* |
| Age | 7.73 | 8.54 | 22.61 | 0.010 |
|  | **PSL** | | | |
|  | **Parametric coefficients** | | | |
| *Term* | *estimate* | *std. error* | *statistic* | *p.value* |
| Intercept | -0.59 | 0.25 | -2.35 | 0.019 |
| Hookworm | -0.44 | 0.40 | -1.11 | 0.269 |
| Malaria | 0.49 | 0.29 | 1.70 | 0.089 |
| Sex | -0.41 | 0.28 | -1.79 | 0.074 |
| *S. mansoni* -KK | 0.11 | 0.23 | 0.38 | 0.704 |
|  | **Smooth terms** | | | |
|  | *edf* | *Ref.df* | *Chi.sq* | *p-value* |
| Age:Malaria=N | 3.51 | 8.04 | 18.07 | 0.022 |
|  | **Anaemia** | | | |
|  | **Parametric coefficients** | | | |
| *Term* | *estimate* | *std. error* | *statistic* | *p.value* |
| Intercept | -2.03 | 0.31 | -6.48 | 0.000 |
| Hookworm | 0.02 | 0.54 | 0.04 | 0.972 |
| Malaria | 0.79 | 0.38 | 2.08 | 0.038 |
| Sex | NA | NA | NA | NA |
| S. mansoni - KK | -0.44 | 0.38 | -1.15 | 0.251 |
|  | **Smooth terms** | | | |
|  | *edf* | *Ref.df* | *Chi.sq* | *p-value* |
| Age | 1.00 | 1.00 | 4.09 | 0.043 |

*PVD=portal vein dilation, PSL=parasternal line, POC-CCA=* *Point-of-care circulating cathodic antigen, std=standard, edf=estimated degrees of freedom, Ref.df=reference degrees of freedom, NA=not applicable – Sex was not included in the anaemia model.*
