# Supplementary material for: The relationship between expelled eggs, morbidity and age in a Schistosoma mansoni endemic setting in Uganda: Implications for current elimination policies
Source: PLoS Negl Trop Dis. 2025 Sep 3;19(9):e0012750. doi: 10.1371/journal.pntd.0012750 (PMC12407471; doi:10.1371/journal.pntd.0012750)
Supplement: S5 Table — (DOCX) [file pntd.0012750.s006.docx]

| **S5 Table. GAM model summaries: *Schistosoma mansoni* infection as measured by POC-CCA as a predictor for portal vein dilation (PVD), enlarged parasternal line (PSL) and anaemia** | | | | |
| --- | --- | --- | --- | --- |
|  | **PVD** | | | |
|  | **Parametric coefficients** | | | |
| *Term* | *estimate* | *std. error* | *statistic* | *p.value* |
| Intercept | -1.47 | 0.31 | -4.72 | <0.001 |
| Hookworm | 0.38 | 0.39 | 1.00 | 0.320 |
| Malaria | 0.57 | 0.30 | 1.91 | 0.056 |
| Sex | 0.52 | 0.28 | 1.86 | 0.062 |
| *S. mansoni* - POC-CCA | 0.46 | 0.32 | 1.44 | 0.149 |
|  | **Smooth terms** | | | |
|  | *edf* | *Ref.df* | *Chi.sq* | *p-value* |
| Age | 4.91 | 5.72 | 18.75 | 0.004 |
|  | **PSL** | | | |
|  | **Parametric coefficients** | | | |
| *Term* | *estimate* | *std. error* | *statistic* | *p.value* |
| Intercept | -0.40 | 0.27 | -1.45 | 0.148 |
| Hookworm | -0.41 | 0.40 | -1.04 | 0.298 |
| Malaria | 0.50 | 0.29 | 1.74 | 0.081 |
| Sex | -0.50 | 0.28 | -1.82 | 0.069 |
| *S. mansoni* - POC-CCA | -0.16 | 0.30 | -0.54 | 0.590 |
|  | **Smooth terms** | | | |
|  | *edf* | *Ref.df* | *Chi.sq* | *p-value* |
| Age | 3.20 | 3.86 | 13.60 | 0.008 |
|  | **Anaemia** | | | |
|  | **Parametric coefficients** | | | |
| *Term* | *estimate* | *std. error* | *statistic* | *p.value* |
| Intercept | -2.20 | 0.40 | -5.46 | <0.001 |
| Hookworm | -0.07 | 0.53 | -0.14 | 0.893 |
| Malaria | 0.73 | 0.38 | 1.92 | 0.055 |
| Sex | 0.31 | 0.37 | 0.85 | 0.396 |
| *S. mansoni* - POC-CCA | -0.24 | 0.41 | -0.59 | 0.553 |
|  | **Smooth terms** | | | |
|  | *edf* | *Ref.df* | *Chi.sq* | *p-value* |
| Age | 1.00 | 1.00 | 3.46 | 0.063 |

*PVD=portal vein dilation, PSL=parasternal line, POC-CCA=* *Point-of-care circulating cathodic antigen, std=standard, edf=estimated degrees of freedom, Ref.df=reference degrees of freedom*
