# Supplementary material for: The relationship between expelled eggs, morbidity and age in a Schistosoma mansoni endemic setting in Uganda: Implications for current elimination policies
Source: PLoS Negl Trop Dis. 2025 Sep 3;19(9):e0012750. doi: 10.1371/journal.pntd.0012750 (PMC12407471; doi:10.1371/journal.pntd.0012750)
Supplement: S6 Table — (DOCX) [file pntd.0012750.s007.docx]

| **S6 Table. GAM model summaries: *Schistosoma mansoni* infection (Kato Katz) as a predictor for self-reported symptoms** | | | | | | | | | |
| --- | --- | --- | --- | --- | --- | --- | --- | --- | --- |
| **Symptom** | **Type** | **Term** | **Estimate** | **Std.Error** | **Statistic** | **p.value** | **edf** | **Ref.df** | **Chi.sq** |
| Abdominal Pain | Parametric | Intercept | 0.919 | 0.273 | 3.364 | 0.001 |  |  |  |
| Abdominal Pain | Parametric | S. mansoni - KK | -0.585 | 0.348 | -1.682 | 0.093 |  |  |  |
| Abdominal Pain | Parametric | Hookworm | -0.547 | 0.442 | -1.238 | 0.216 |  |  |  |
| Abdominal Pain | Parametric | Malaria | -0.008 | 0.353 | -0.022 | 0.982 |  |  |  |
| Abdominal Pain | Smooth | Age |  |  |  | 0.018 | 7.568 | 8.427 | 19.520 |
| Blood in Stool | Parametric | Intercept | -3.600 | 4.157 | -0.866 | 0.386 |  |  |  |
| Blood in Stool | Parametric | S. mansoni - KK | 0.214 | 0.472 | 0.453 | 0.650 |  |  |  |
| Blood in Stool | Parametric | Hookworm | -0.895 | 0.677 | -1.322 | 0.186 |  |  |  |
| Blood in Stool | Parametric | Malaria | 0.312 | 0.423 | 0.739 | 0.460 |  |  |  |
| Blood in Stool | Smooth | Age |  |  |  | 0.351 | 9.574 | 10.810 | 12.380 |
| Body Swelling | Parametric | Intercept | -2.699 | 0.476 | -5.677 | 0.000 |  |  |  |
| Body Swelling | Parametric | S. mansoni - KK | 0.170 | 0.587 | 0.290 | 0.772 |  |  |  |
| Body Swelling | Parametric | Hookworm | -32.190 | 1.27E+07 | 0.000 | 1.000 |  |  |  |
| Body Swelling | Parametric | Malaria | 0.362 | 0.613 | 0.590 | 0.555 |  |  |  |
| Body Swelling | Smooth | Age |  |  |  | 0.747 | 1.444 | 1.766 | 0.342 |
| Chills | Parametric | Intercept | -1.040 | 0.378 | -2.756 | 0.006 |  |  |  |
| Chills | Parametric | S. mansoni - KK | -0.571 | 0.406 | -1.407 | 0.159 |  |  |  |
| Chills | Parametric | Hookworm | -0.111 | 0.508 | -0.217 | 0.828 |  |  |  |
| Chills | Parametric | Malaria | 0.201 | 0.386 | 0.522 | 0.602 |  |  |  |
| Chills | Smooth | Age |  |  |  | 0.005 | 6.611 | 7.588 | 22.380 |
| Diarrhoea | Parametric | Intercept | -0.083 | 0.241 | -0.342 | 0.732 |  |  |  |
| Diarrhoea | Parametric | S. mansoni - KK | -0.126 | 0.310 | -0.406 | 0.685 |  |  |  |
| Diarrhoea | Parametric | Hookworm | -0.762 | 0.459 | -1.662 | 0.097 |  |  |  |
| Diarrhoea | Parametric | Malaria | -0.067 | 0.326 | -0.204 | 0.838 |  |  |  |
| Diarrhoea | Smooth | Age |  |  |  | 0.003 | 1.000 | 1.000 | 9.069 |
| Difficulty Breathing | Parametric | Intercept | -2.464 | 0.466 | -5.287 | 0.000 |  |  |  |
| Difficulty Breathing | Parametric | S. mansoni - KK | -1.041 | 0.727 | -1.434 | 0.152 |  |  |  |
| Difficulty Breathing | Parametric | Hookworm | -44.610 | 1.27E+07 | 0.000 | 1.000 |  |  |  |
| Difficulty Breathing | Parametric | Malaria | 0.184 | 0.768 | 0.239 | 0.811 |  |  |  |
| Difficulty Breathing | Smooth | Age |  |  |  | 0.932 | 1.000 | 1.001 | 0.007 |
| Dizziness | Parametric | Intercept | -0.456 | 0.256 | -1.780 | 0.075 |  |  |  |
| Dizziness | Parametric | S. mansoni - KK | 0.187 | 0.334 | 0.562 | 0.574 |  |  |  |
| Dizziness | Parametric | Hookworm | 0.206 | 0.433 | 0.476 | 0.634 |  |  |  |
| Dizziness | Parametric | Malaria | -0.482 | 0.351 | -1.373 | 0.170 |  |  |  |
| Dizziness | Smooth | Age |  |  |  | 0.015 | 3.109 | 3.881 | 12.070 |
| Fever | Parametric | Intercept | -0.092 | 0.298 | -0.308 | 0.758 |  |  |  |
| Fever | Parametric | S. mansoni - KK | -0.156 | 0.338 | -0.463 | 0.643 |  |  |  |
| Fever | Parametric | Hookworm | -0.271 | 0.434 | -0.625 | 0.532 |  |  |  |
| Fever | Parametric | Malaria | 0.078 | 0.336 | 0.233 | 0.816 |  |  |  |
| Fever | Smooth | Age |  |  |  | 0.072 | 7.639 | 8.481 | 16.180 |
| Headache | Parametric | Intercept | 0.824 | 0.309 | 2.670 | 0.008 |  |  |  |
| Headache | Parametric | S. mansoni - KK | 0.216 | 0.351 | 0.615 | 0.539 |  |  |  |
| Headache | Parametric | Hookworm | -0.605 | 0.423 | -1.428 | 0.153 |  |  |  |
| Headache | Parametric | Malaria | 0.214 | 0.359 | 0.597 | 0.551 |  |  |  |
| Headache | Smooth | Age |  |  |  | 0.250 | 1.019 | 1.037 | 1.327 |
| Muscle Pain | Parametric | Intercept | -0.977 | 0.352 | -2.775 | 0.006 |  |  |  |
| Muscle Pain | Parametric | S. mansoni - KK | -0.511 | 0.398 | -1.285 | 0.199 |  |  |  |
| Muscle Pain | Parametric | Hookworm | 0.270 | 0.523 | 0.516 | 0.606 |  |  |  |
| Muscle Pain | Parametric | Malaria | -0.921 | 0.531 | -1.736 | 0.083 |  |  |  |
| Muscle Pain | Smooth | Age |  |  |  | 0.002 | 1.659 | 2.067 | 13.090 |
| Nausea | Parametric | Intercept | -1.063 | 0.326 | -3.262 | 0.001 |  |  |  |
| Nausea | Parametric | S. mansoni - KK | -0.140 | 0.363 | -0.386 | 0.700 |  |  |  |
| Nausea | Parametric | Hookworm | 0.081 | 0.444 | 0.182 | 0.856 |  |  |  |
| Nausea | Parametric | Malaria | 0.619 | 0.353 | 1.751 | 0.080 |  |  |  |
| Nausea | Smooth | Age |  |  |  | 0.035 | 2.589 | 3.186 | 9.021 |
| Pain During Urination | Parametric | Intercept | -1.212 | 0.371 | -3.262 | 0.001 |  |  |  |
| Pain During Urination | Parametric | S. mansoni - KK | 0.437 | 0.406 | 1.076 | 0.282 |  |  |  |
| Pain During Urination | Parametric | Hookworm | -0.515 | 0.503 | -1.024 | 0.306 |  |  |  |
| Pain During Urination | Parametric | Malaria | -0.318 | 0.403 | -0.789 | 0.430 |  |  |  |
| Pain During Urination | Smooth | Age |  |  |  | 0.000 | 4.319 | 5.308 | 29.400 |
| Rash | Parametric | Intercept | -2.017 | 0.443 | -4.551 | 0.000 |  |  |  |
| Rash | Parametric | S. mansoni - KK | 0.863 | 0.478 | 1.807 | 0.071 |  |  |  |
| Rash | Parametric | Hookworm | -0.279 | 0.523 | -0.534 | 0.593 |  |  |  |
| Rash | Parametric | Malaria | -0.099 | 0.406 | -0.244 | 0.807 |  |  |  |
| Rash | Smooth | Age |  |  |  | 0.008 | 8.525 | 8.919 | 22.180 |
| Vomiting | Parametric | Intercept | -1.510 | 0.363 | -4.157 | 0.000 |  |  |  |
| Vomiting | Parametric | S. mansoni - KK | -0.030 | 0.403 | -0.073 | 0.942 |  |  |  |
| Vomiting | Parametric | Hookworm | -0.093 | 0.505 | -0.183 | 0.854 |  |  |  |
| Vomiting | Parametric | Malaria | 0.632 | 0.368 | 1.718 | 0.086 |  |  |  |
| Vomiting | Smooth | Age |  |  |  | 0.199 | 2.440 | 3.068 | 4.763 |
| Weakness | Parametric | Intercept | -0.678 | 0.301 | -2.252 | 0.024 |  |  |  |
| Weakness | Parametric | S. mansoni - KK | -0.406 | 0.386 | -1.052 | 0.293 |  |  |  |
| Weakness | Parametric | Hookworm | -0.764 | 0.529 | -1.445 | 0.149 |  |  |  |
| Weakness | Parametric | Malaria | -0.230 | 0.414 | -0.556 | 0.578 |  |  |  |
| Weakness | Smooth | Age |  |  |  | 0.002 | 6.837 | 7.893 | 24.330 |
