# Supplementary material for: The relationship between expelled eggs, morbidity and age in a Schistosoma mansoni endemic setting in Uganda: Implications for current elimination policies
Source: PLoS Negl Trop Dis. 2025 Sep 3;19(9):e0012750. doi: 10.1371/journal.pntd.0012750 (PMC12407471; doi:10.1371/journal.pntd.0012750)
Supplement: S7 Table — (DOCX) [file pntd.0012750.s008.docx]

| **S7 Table. GAM model summaries: *Schistosoma mansoni* infection (POC-CCA) as a predictor for self-reported symptoms** | | | | | | | | | |
| --- | --- | --- | --- | --- | --- | --- | --- | --- | --- |
| **Symptom** | **Type** | **Term** | **Estimate** | **Std.Error** | **Statistic** | **p.value** | **edf** | **Ref.df** | **Chi.sq** |
| Abdominal Pain | Parametric | Intercept | 0.710 | 0.310 | 2.289 | 0.022 |  |  |  |
| Abdominal Pain | Parametric | S. mansoni - POC-CCA | -0.150 | 0.344 | -0.438 | 0.662 |  |  |  |
| Abdominal Pain | Parametric | Hookworm | -0.668 | 0.435 | -1.537 | 0.124 |  |  |  |
| Abdominal Pain | Parametric | Malaria | -0.035 | 0.350 | -0.100 | 0.920 |  |  |  |
| Abdominal Pain | Smooth | Age |  |  |  | 0.046 | 7.587 | 8.441 | 16.740 |
| Blood in Stool | Parametric | Intercept | -6.958 | 10.886 | -0.639 | 0.523 |  |  |  |
| Blood in Stool | Parametric | S. mansoni - POC-CCA | 1.591 | 0.694 | 2.293 | 0.022 |  |  |  |
| Blood in Stool | Parametric | Hookworm | -1.188 | 0.725 | -1.639 | 0.101 |  |  |  |
| Blood in Stool | Parametric | Malaria | 0.785 | 0.493 | 1.591 | 0.112 |  |  |  |
| Blood in Stool | Smooth | Age |  |  |  | 0.036 | 13.97 | 15.57 | 28.620 |
| Body Swelling | Parametric | Intercept | -2.228 | 0.499 | -4.470 | 0.000 |  |  |  |
| Body Swelling | Parametric | S. mansoni - POC-CCA | -0.605 | 0.592 | -1.023 | 0.306 |  |  |  |
| Body Swelling | Parametric | Hookworm | -39.120 | 1.27E+07 | 0.000 | 1.000 |  |  |  |
| Body Swelling | Parametric | Malaria | 0.386 | 0.622 | 0.620 | 0.535 |  |  |  |
| Body Swelling | Smooth | Age |  |  |  | 0.690 | 1.582 | 1.967 | 0.712 |
| Chills | Parametric | Intercept | -0.925 | 0.417 | -2.219 | 0.027 |  |  |  |
| Chills | Parametric | S. mansoni - POC-CCA | -0.610 | 0.403 | -1.513 | 0.130 |  |  |  |
| Chills | Parametric | Hookworm | -0.226 | 0.497 | -0.455 | 0.649 |  |  |  |
| Chills | Parametric | Malaria | 0.156 | 0.384 | 0.406 | 0.685 |  |  |  |
| Chills | Smooth | Age |  |  |  | 0.004 | 6.592 | 7.553 | 22.660 |
| Diarrhoea | Parametric | Intercept | -0.099 | 0.293 | -0.336 | 0.737 |  |  |  |
| Diarrhoea | Parametric | S. mansoni - POC-CCA | -0.070 | 0.330 | -0.213 | 0.831 |  |  |  |
| Diarrhoea | Parametric | Hookworm | -0.793 | 0.452 | -1.757 | 0.079 |  |  |  |
| Diarrhoea | Parametric | Malaria | -0.075 | 0.325 | -0.232 | 0.817 |  |  |  |
| Diarrhoea | Smooth | Age |  |  |  | 0.003 | 1 | 1 | 8.716 |
| Difficulty Breathing | Parametric | Intercept | -2.378 | 0.547 | -4.351 | 0.000 |  |  |  |
| Difficulty Breathing | Parametric | S. mansoni - POC-CCA | -0.830 | 0.684 | -1.213 | 0.225 |  |  |  |
| Difficulty Breathing | Parametric | Hookworm | -42.860 | 1.27E+07 | 0.000 | 1.000 |  |  |  |
| Difficulty Breathing | Parametric | Malaria | 0.106 | 0.757 | 0.140 | 0.889 |  |  |  |
| Difficulty Breathing | Smooth | Age |  |  |  | 0.960 | 1.002 | 1.004 | 0.004 |
| Fever | Parametric | Intercept | -0.092 | 0.298 | -0.308 | 0.758 |  |  |  |
| Fever | Parametric | S. mansoni - POC-CCA | -0.156 | 0.338 | -0.463 | 0.643 |  |  |  |
| Fever | Parametric | Hookworm | -0.271 | 0.434 | -0.625 | 0.532 |  |  |  |
| Fever | Parametric | Malaria | 0.078 | 0.336 | 0.233 | 0.816 |  |  |  |
| Fever | Smooth | Age |  |  |  | 0.072 | 7.639 | 8.481 | 16.180 |
| Headache | Parametric | Intercept | 0.824 | 0.309 | 2.670 | 0.008 |  |  |  |
| Headache | Parametric | S. mansoni - POC-CCA | 0.216 | 0.351 | 0.615 | 0.539 |  |  |  |
| Headache | Parametric | Hookworm | -0.605 | 0.423 | -1.428 | 0.153 |  |  |  |
| Headache | Parametric | Malaria | 0.214 | 0.359 | 0.597 | 0.551 |  |  |  |
| Headache | Smooth | Age |  |  |  | 0.250 | 1.019 | 1.037 | 1.327 |
| Muscle Pain | Parametric | Intercept | -0.977 | 0.352 | -2.775 | 0.006 |  |  |  |
| Muscle Pain | Parametric | S. mansoni - POC-CCA | -0.511 | 0.398 | -1.285 | 0.199 |  |  |  |
| Muscle Pain | Parametric | Hookworm | 0.270 | 0.523 | 0.516 | 0.606 |  |  |  |
| Muscle Pain | Parametric | Malaria | -0.921 | 0.531 | -1.736 | 0.083 |  |  |  |
| Muscle Pain | Smooth | Age |  |  |  | 0.002 | 1.659 | 2.067 | 13.090 |
| Nausea | Parametric | Intercept | -1.063 | 0.326 | -3.262 | 0.001 |  |  |  |
| Nausea | Parametric | S. mansoni - POC-CCA | -0.140 | 0.363 | -0.386 | 0.700 |  |  |  |
| Nausea | Parametric | Hookworm | 0.081 | 0.444 | 0.182 | 0.856 |  |  |  |
| Nausea | Parametric | Malaria | 0.619 | 0.353 | 1.751 | 0.080 |  |  |  |
| Nausea | Smooth | Age |  |  |  | 0.035 | 2.589 | 3.186 | 9.021 |
| Pain During Urination | Parametric | Intercept | -1.212 | 0.371 | -3.262 | 0.001 |  |  |  |
| Pain During Urination | Parametric | S. mansoni - POC-CCA | 0.437 | 0.406 | 1.076 | 0.282 |  |  |  |
| Pain During Urination | Parametric | Hookworm | -0.515 | 0.503 | -1.024 | 0.306 |  |  |  |

| Pain During Urination | Parametric | Malaria | -0.318 | 0.403 | -0.789 | 0.430 |  |  |  |
| --- | --- | --- | --- | --- | --- | --- | --- | --- | --- |
| Pain During Urination | Smooth | Age |  |  |  | 0.000 | 4.319 | 5.308 | 29.400 |
| Rash | Parametric | Intercept | -2.017 | 0.443 | -4.551 | 0.000 |  |  |  |
| Rash | Parametric | S. mansoni - POC-CCA | 0.863 | 0.478 | 1.807 | 0.071 |  |  |  |
| Rash | Parametric | Hookworm | -0.279 | 0.523 | -0.534 | 0.593 |  |  |  |
| Rash | Parametric | Malaria | -0.099 | 0.406 | -0.244 | 0.807 |  |  |  |
| Rash | Smooth | Age |  |  |  | 0.008 | 8.525 | 8.919 | 22.180 |
| Vomiting | Parametric | Intercept | -1.510 | 0.363 | -4.157 | 0.000 |  |  |  |
| Vomiting | Parametric | S. mansoni - POC-CCA | -0.030 | 0.403 | -0.073 | 0.942 |  |  |  |
| Vomiting | Parametric | Hookworm | -0.093 | 0.505 | -0.183 | 0.854 |  |  |  |
| Vomiting | Parametric | Malaria | 0.632 | 0.368 | 1.718 | 0.086 |  |  |  |
| Vomiting | Smooth | Age |  |  |  | 0.199 | 2.44 | 3.068 | 4.763 |
| Weakness | Parametric | Intercept | -0.622 | 0.335 | -1.860 | 0.063 |  |  |  |
| Weakness | Parametric | S. mansoni - POC-CCA | -0.277 | 0.375 | -0.738 | 0.461 |  |  |  |
| Weakness | Parametric | Hookworm | -0.765 | 0.520 | -1.472 | 0.141 |  |  |  |
| Weakness | Parametric | Malaria | -0.251 | 0.401 | -0.624 | 0.532 |  |  |  |
| Weakness | Smooth | Age |  |  |  | 0.000 | 3.769 | 4.667 | 23.610 |
